# Alcohol consumption is associated with an increased risk of erosive esophagitis and Barrett's epithelium in Japanese men

**DOI:** 10.1186/1471-230X-8-58

**Published:** 2008-12-11

**Authors:** Tomoyuki Akiyama, Masahiko Inamori, Hiroshi Iida, Hironori Mawatari, Hiroki Endo, Kunihiro Hosono, Kyoko Yoneda, Koji Fujita, Masato Yoneda, Hirokazu Takahashi, Ayumu Goto, Yasunobu Abe, Noritoshi Kobayashi, Kensuke Kubota, Satoru Saito, Atsushi Nakajima

**Affiliations:** 1 Gastroenterology Division, Yokohama City University School of Medicine, Japan

## Abstract

**Background:**

Evidence regarding the association between alcohol consumption and the gastro-esophageal reflux disease (GERD) spectrum has been conflicting. We examined the association between alcohol consumption and erosive esophagitis and Barrett's epithelium in Japanese men.

**Methods:**

The study population comprised 463 men subjects who had undergone an upper endoscopy at the Gastroenterology Division of Yokohama City University Hospital between August 2005 and July 2006. The presence of erosive esophagitis and Barrett's epithelium was diagnosed based on the Los Angeles Classification and the Prague C and M Criteria, respectively. We divided the study population into four groups: never drinkers, light drinkers (less than 25.0 g of ethanol per day), moderate drinkers (25.0 to 50.0 g of ethanol per day), and heavy drinkers (more than 50.0 g of ethanol per day). A linear regression of the logistic regression analysis was used to analyze the dose-response trends.

**Results:**

Compared with never drinkers, light drinkers (less than 25.0 g ethanol per day), moderate drinkers (25.0 to 50.0 g per day), and heavy drinkers (more than 50.0 g per day) had ORs for erosive esophagitis of 1.110 (95% CI: 0.553 – 2.228, p = 0.7688), 1.880 (95% CI: 1.015 – 3.484, p = 0.0445) and 1.988 (95% CI: 1.120 – 3.534, p = 0.0190), respectively. These groups had ORs for Barrett's epithelium of 1.278 (95% CI: 0.752 – 2.170, p = 0.3643), 1.458 (95% CI: 0.873 – 2.433, p = 0.1500), and 1.912 (95% CI: 1.185 – 3.086, p = 0.0079), respectively. The odds ratios/grams (alcohol)/day of dose response trends for erosive esophagitis and Barrett's epithelium were 1.015 (95% CI: 1.004–1.026, p = 0.0066) and 1.012 (95% CI: 1.003–1.021, p = 0.0079), respectively.

**Conclusion:**

These findings suggest that alcohol consumption in Japanese men tends to be associated with an increased risk of erosive esophagitis and Barrett's epithelium.

## Background

In cases with Barrett's epithelium, the resulting replacement of normal squamous epithelium with columnar epithelium can be seen in the distal esophagus as a salmon-pink colored area that is readily visible during endoscopic examinations. The proximal level of the squamocolumnar junction no longer coincides with the gastroesophageal junction in cases with Barrett's epithelium. Barrett's epithelium, which is recognized as a complication of erosive esophagitis, is recognized as a pre-malignant condition that may lead to the development of esophageal adenocarcinoma [1,2]. The incidence of esophageal adenocarcinoma is rapidly increasing in North America and Europe [3-5]. For esophageal carcinoma in Japan, however, the ratio of adenocarcinoma to squamous cell carcinoma is low, and no significant changes have been identified [6]. As the prevalence of erosive esophagitis is increasing, further observation of Barrett's epithelium is required in Japan.

Although a strong statistical association between erosive esophagitis and a risk of Barrett's epithelium has been reported [7-10], the roles of lifestyle risk factors, including alcohol consumption, are less well defined and remain controversial [11-15]. Therefore, we examined the association between alcohol consumption and the risk of erosive esophagitis and Barrett's epithelium in a retrospective cohort study of Japanese men.

## Methods

### Patients

A total of 463 male subjects (median age, 67 years; age range, 31 – 91 years) who had undergone an upper endoscopy at the Gastroenterology Division of Yokohama City University Hospital between August 2005 and July 2006 were enrolled in the present study. The total study population had undergone endoscopies as part of health checkups, and the majority of these subjects were outpatients. Cases were excluded if their complete profiles could not be obtained from their medical records, if they refused to participate in the present study, or if they had previously undergone an upper digestive tract operation.

The computerized endoscopic records were retrieved for the entire study population, and all of the endoscopic films were reviewed by two trained endoscopists. Hiatal hernia was diagnosed when the distance between the gastroesophageal junction and the diaphragmatic hiatus was 2 cm or more. Erosive esophagitis was diagnosed based on the Los Angeles Classification [16] and was divided into three groups: none, mild (grades A and B), or severe (grades C and D). The presence of Barrett's epithelium was diagnosed based on the Prague C & M Criteria [17]. According to the criteria, Barrett's epithelium is defined as the macroscopic identification, using a standard endoscopy exam, of abnormal columnar esophageal epithelium suggestive of columnar-lined distal esophagus. The length of Barrett's epithelium is measured using the circumferential extent (the C value) and the maximum extent (the M value) above the gastroesophageal junction, identified as the proximal margin of the gastric mucosal folds in centimeters [17].

Complete patient information, including age, sex, body mass index (BMI), regular drinking habit, and smoking habit at the time of the initial diagnosis was obtained from each patient's medical records. "Regular drinking habit" was defined in this study as a current regular drinker. Regular drinkers were asked about the frequency of their drinking habit (once or twice per week, three or four times per week, or five times or more per week), the amount drunk on each occasion, and the types of beverage usually consumed (sake, shochu, beer, whisky, wine, or others). "Shochu" is a clear liquor commonly distilled from sweet potatoes, rice, or buckwheat. From these data, we calculated the amount of ethanol (in grams) consumed per day according to the standard ethanol percentage: sake (15%), shochu (25%), beer (5%), whisky (40%), and wine (15%). The study population was then classified into four groups: never drinkers, light drinkers (less than 25.0 g of ethanol per day), moderate drinkers (25.0 to 50.0 g of ethanol per day), and heavy drinkers (more than 50.0 g of ethanol per day). "Smoking habit" was defined in this study as a current regular smoker.

### Ethics

The study was conducted in accordance with the Declaration of Helsinki. The study protocol was approved by the Ethics Committee of Yokohama City University Hospital. All the patients provided their written informed consent.

### Statistical analysis

We used a logistic regression analysis to estimate the odds ratio (OR) and 95% confidence interval (CI) for the incidence of erosive esophagitis and Barrett's epithelium according to drinking status. The analysis was performed using Stat View software (SAS Institute, Cary, N.C.). As the primary outcome, we examined the association between alcohol consumption and the respective risks of erosive esophagitis and Barrett's epithelium. The odds ratios were computed as the incidence rate among the subjects for each status of alcohol consumption divided by the rate among those who had never drunk alcohol. The subjects who had never drunk alcohol were treated as a reference group. The p – values for the analysis of linear trends were also calculated by assigning ordinal exposure variables as continuous terms for the volume of alcohol drunk per day (never drinkers were coded as zero). A linear regression of the logistic regression analysis was used to analyze the dose-response trends. All p – values were two-tailed, and a p value < 0.05 was considered statistically significant.

## Results

The baseline characteristics of the study population are summarized in Additional file 1. At baseline, the proportions of never drinkers, light drinkers, moderate drinkers, and heavy drinkers were 40.4%, 17.3%, 18.8%, and 23.5%, respectively. Alcohol consumption is socio-culturally acceptable in Japan, with alcohol being widely consumed as the most popular beverage among adults, and only a few people actually abstain from drinking alcohol. As a result, the proportion of never drinkers was smaller than the overall proportion of regular drinkers in this study population.

A total of 97 cases (21.0%) had erosive esophagitis: 90 cases (19.4%) had mild esophagitis (LA grades A and B), and 7 cases (1.5%) had severe esophagitis (LA grades C and D) (Additional file 1). The prevalence of erosive esophagitis, previously believed to be relatively low in Asian countries, including Japan, appears to be increasing. The prevalence of esophagitis in Japan was 3% in the 1970s [18], increased to 10–15% in the late 1990s [19], and the present study in 2006 showed a prevalence of 21.0%; most of these cases were mild esophagitis (19.4%), and severe esophagitis was relatively uncommon (1.5%) (Additional file 1).

Additional file 2 compares the baseline lifestyle characteristics of never drinkers and regular drinkers. Compared to subjects who had never drunk alcohol, regular drinkers were older, less likely to be obese (body mass index > 25), and more likely to be smokers. In a univariate analysis, however, only a statistically significant difference in smoking habit was observed between the two groups (Additional file 2). The patient profiles for each group of regular drinkers were as follows: never drinkers (n = 187; mean age, 64 years; mean BMI, 22.8; smoking habit, 50.3%), light drinkers (n = 80; mean age, 65 years; mean BMI, 22.4; smoking habit, 51.3%), moderate drinkers (n = 87; mean age, 68 years; mean BMI, 23.0; smoking habit, 65.5%), and heavy drinkers (n = 109; mean age, 65 years; mean BMI, 23.1; smoking habit, 66.1%).

Additional file 3 presents the odds ratios (ORs) and 95% confidence intervals (CIs) for hiatal hernia, erosive esophagitis, and Barrett's epithelium according to the different alcohol consumption levels. Compared with never drinkers, light drinkers (less than 25.0 g ethanol per day), moderate drinkers (25.0 to 50.0 g per day), and heavy drinkers (more than 50.0 g per day) had ORs for erosive esophagitis of 1.110 (95% CI: 0.553 – 2.228, p = 0.7688), 1.880 (95% CI: 1.015 – 3.484, p = 0.0445) and 1.988 (95% CI: 1.120 – 3.534, p = 0.0190), respectively. These groups had ORs for Barrett's epithelium of 1.278 (95% CI: 0.752 – 2.170, p = 0.3643), 1.458 (95% CI: 0.873 – 2.433, p = 0.1500), and 1.912 (95% CI: 1.185 – 3.086, p = 0.0079), respectively. The odds ratios/grams (alcohol)/day of dose response trends for erosive esophagitis and Barrett's epithelium were 1.015 (95% CI: 1.004–1.026, p = 0.0066) and 1.012 (95% CI: 1.003–1.021, p = 0.0079), respectively.

## Discussion

The present study demonstrated that 45.6% of the total study population was diagnosed as having Barrett's epithelium, based on the Prague C & M Criteria [17]. These cases consisted of 45.1% with short-segment Barrett's esophagus (SSBE), the circumferential (C) extent of which was less than 3 cm, and 0.4% with long-segment Barrett's esophagus (LSBE), the C extent of which was 3 cm or more (Additional file 1). These figures are consistent with those of a previous Japanese report that concluded that the incidence of SSBE was higher in Japan than in the United States and Western Europe, whereas LSBE was much rarer (Additional file 1) [20]. The low incidence of esophageal adenocarcinoma in the Japanese population, despite the high incidence of Barrett's epithelium in the study population, is an unusual phenomenon. The frequency of Barrett's epithelium might be affected by whether its definition requires the histological confirmation of specialized intestinal metaplasia. The British Society of Gastroenterology guidelines have shown that the histological evidence of specialized intestinal metaplasia is not necessary for the diagnosis of Barrett's esophagus, as its absence on one set of biopsies may be solely due to a sampling error, and the tissue may still have an increased neoplastic potential compared with squamous-lined esophagus [21]. This conclusion is regarded as being valid. In western countries except U.K., however, the confirmation of intestinal metaplasia of the esophagus based on biopsy results is thought to be essential for the diagnosis of Barrett's epithelium [22], as it is considered a risk factor for esophageal adenocarcinoma [23]. In the present study, Barrett's epithelium was diagnosed endoscopically based on the Prague C & M Criteria [17], without requiring histological confirmation; thus, the patients in this study were actually diagnosed as having endoscopic Barrett's epithelium. In addition, as the prevalence of esophageal adenocarcinoma is not as high among non-Caucasians in the United States as it is among Caucasians [24], large ethnic differences in the pathogenesis process of esophageal adenocarcinoma may exist.

Additional file 3 suggested that alcohol consumption in Japanese men tends to be associated with an increased risk of erosive esophagitis and Barrett's epithelium. In contrast, no association was found between the volume of alcohol drunk per day and the increased risk of hiatal hernia (p for trend = 0.8121) (Additional file 3). Additionally, these findings suggest that alcohol consumption may be more likely to contribute to the development of erosive esophagitis than Barrett's epithelium.

Although several cohort studies have examined the risk of gastroesophageal reflux disease (GERD) in relation to alcohol consumption, some aspects remain controversial. Some authors believed that alcohol use was a risk factor for GERD [25], whereas others suggested that alcohol exposure was not associated with the risk of GERD [26]. In the present population-based retrospective cohort study of Japanese men, we examined the association between alcohol consumption and the risk of erosive esophagitis and Barrett's epithelium, which are complications of GERD. The present study showed that alcohol consumption in Japanese men tends to be associated with an increased risk of erosive esophagitis and Barrett's epithelium (Additional file 3).

The consumption of large amounts of alcohol with a normal meal has been reported to facilitate acid regurgitation by reducing the pressure of the lower esophageal sphincter and slowing both esophageal motility and gastric emptying [27,28]. In chronic alcoholics, gastroesophageal reflux is often caused by esophageal peristaltic dysfunction, with an increase in the amplitude of the contractions in the middle third of the esophagus and a decrease in the lower/middle amplitude ratio [29]. Likewise, a decrease in salivary bicarbonate and peripheral neuropathy affecting muscle contraction have also been suggested as additional factors that may contribute to the reduction in acid clearance in the esophagus [27]. Alcohol not only causes direct damage to the esophageal mucosa, but also has noxious effects once it is no longer in contact with the mucosa. The pathophysiological mechanisms by which alcohol damages the epithelium include alterations in epithelial transport, intercellular junction disorders, and impairment of the mucosal barrier-all of which, in turn, have been reported to facilitate hydrogen ion penetration into the mucosa [30]. Regardless of the type of alcoholic beverage involved (liquors, white wine, or beer), lower alcohol doses also have been shown to induce pressure decrements in both the upper and lower esophageal sphincters, as well as a decrease in esophageal motility and an enhanced risk of gastroesophageal reflux in healthy volunteers [31,32]. Further physiological studies examining differences among patients who consume alcohol are necessary for investigating the influence of alcohol consumption on the mechanism of erosive esophagitis and Barrett's epithelium.

Our study had several limitations that may need to be considered. First, information on alcohol drinking and other variables were collected using self-administered questionnaires; thus, some misclassification of the subjects may be inevitable. Second, different volumes of specific beverage types were not identified, and we were unable to conduct a separate analysis for these subgroups. Veugelers et al. reported that increased liquor consumption was a risk factor for both GERD and Barrett's esophagus [33]. Third, only men were examined in our study population. Whether our results are also applicable to women remains to be determined. Finally, we could not include several confounding variables, such as the use of non-steroidal anti-inflammatory drugs (NSAIDs) or aspirin. Aspirin and other NSAIDs have been found to be associated with esophagitis and esophageal strictures in some studies [34,35], but such associations have not been identified in other studies [34,36].

## Conclusion

In conclusion, our data suggests that alcohol consumption in Japanese men tends to be associated with an increased risk of erosive esophagitis and Barrett's epithelium. From a public health perspective, further studies are needed to clarify the role of alcohol consumption in the etiology of erosive esophagitis and Barrett's epithelium.

## Abbreviations

BMI: body mass index; OR: odds ratio; CI: confidence interval; LA: the Los Angeles Classification; C extent: circumferential extent; M extent: maximum extent; SSBE: short-segment Barrett's esophagus; LSBE: long-segment Barrett's esophagus.

## Competing interests

The authors declare that they have no competing interests.

## Authors' contributions

TA analyzed the upper endoscopies, collected the clinical data and wrote the manuscript, with contributions from MI. AN was responsible for the design of the study and collected the clinical data. MI performed the statistical analyses. TA and MI analyzed the upper endoscopies and participated in the design and coordination of the study. All authors read and approved the final manuscript.

## Pre-publication history

The pre-publication history for this paper can be accessed here:



## Supplementary Material

Additional file 1 **Table 1. Clinical characteristics of subjects enrolled in the present study.** Click here for file

Additional file 2 **Table 2. Lifestyle characteristics of never drinkers and regular drinkers** Click here for file

Additional file 3 **Table 3. Odds ratios (ORs), 95% confidence interval (CI) and P value of hiatal hernia, erosive esophagitis, and Barrett’s epithelium according to different alcohol consumption levels.** *logistic regression for analysis. **linear regression of logistic regression for analysis of dose-response trends. Click here for file
