# Supplementary material for: Alcohol consumption is associated with an increased risk of erosive esophagitis and Barrett's epithelium in Japanese men
Source: BMC Gastroenterol. 2008 Dec 11;8:58. doi: 10.1186/1471-230X-8-58 (PMC2615024; doi:10.1186/1471-230X-8-58)
Supplement: Additional file 1 — Table 1. Clinical characteristics of subjects enrolled in the present study. [file 1471-230X-8-58-S1.doc]

| Clinical characteristics Number (%) |
| --- |
| Total patients 463  Patient profiles  Age median; range (years) 67 (31-91)  Body mass index > 25 109 (23.5)  Regular drinking habit  Total 276 (59.6)  Light (< 25 g) 80 (17.3)  Moderate (25 – 50 g) 87 (18.8)  Heavy (> 50 g) 109 (23.5)  Current smoking habit 264 (57.0)  Endoscopic results  Hiatal hernia 146 (31.5)  Erosive esophagitis  Total 97 (21.0)  Mild 90 (19.4)  Severe 7 (1.5)  Barrett’s epithelium  Total 211 (45.6)  SSBE 209 (45.1)  LSBE 2 (0.4) |

Table 1. Clinical characteristics of subjects enrolled in the present study.

SSBE, short-segment Barrett’s esophagus; LSBE, long-segment Barrett’s esophagus
