# Supplementary material for: Alcohol consumption is associated with an increased risk of erosive esophagitis and Barrett's epithelium in Japanese men
Source: BMC Gastroenterol. 2008 Dec 11;8:58. doi: 10.1186/1471-230X-8-58 (PMC2615024; doi:10.1186/1471-230X-8-58)
Supplement: Additional file 2 — Table 2. Lifestyle characteristics of never drinkers and regular drinkers [file 1471-230X-8-58-S2.doc]

| Lifestyle characteristics | Never drinkers  n=187  Number (%) | Regular drinkers  n=276  Number (%) | P-value |
| --- | --- | --- | --- |
| Age median; range (years) | 66 (31-91) | 67 (31-86) | 0.2307 |
| Body mass index > 25 | 46 (24.6) | 63 (22.8) | 0.6591 |
| Smoking habit | 94 (50.3) | 170 (61.6) | 0.0157 |

Table 2. Lifestyle characteristics of never drinkers and regular drinkers
