# Supplementary material for: Alcohol consumption is associated with an increased risk of erosive esophagitis and Barrett's epithelium in Japanese men
Source: BMC Gastroenterol. 2008 Dec 11;8:58. doi: 10.1186/1471-230X-8-58 (PMC2615024; doi:10.1186/1471-230X-8-58)
Supplement: Additional file 3 — Table 3. Odds ratios (ORs), 95% confidence interval (CI) and P value of hiatal hernia, erosive esophagitis, and Barrett’s epithelium according to different alcohol consumption levels. *logistic regression for analysis. **linear regression of logistic regression for analysis of dose-response trends. [file 1471-230X-8-58-S3.doc]

| Endoscopic results | Alcohol consumption (g/day) | | | | Dose response trends  OR  (95%CI)  P value ** |
| --- | --- | --- | --- | --- | --- |
|  | Never drinkers | Regular drinkers | | |  |
|  |  | Light* | Moderate* | Heavy* |  |
|  |  | < 25.0 | 25.0 – 50.0 | > 50.0 |  |
| Hiatal hernia | 1.00 (Referent) | 0.672  (0.372- 1.213)  0.1872 | 0.907  (0.525-1.567)  0.7272 | 1.036  (0.629-1.709)  0.8895 | 1.001  (0.992-1.011)  0.8121 |
| Erosive esophagitis | 1.00 (Referent) | 1.110  (0.553-2.228)  0.7688 | 1.880  (1.015-3.484)  0.0445 | 1.988  (1.120-3.534)  0.0190 | 1.015  (1.004-1.026)  0.0066 |
| Barrett’s epithelium | 1.00 (Referent) | 1.278  (0.752-2.170)  0.3643 | 1.458  (0.873-2.433)  0.1500 | 1.912  (1.185-3.086)  0.0079 | 1.012  (1.003-1.0021)  0.0079 |

Table 3. Odds ratios (ORs), 95% confidence interval (CI) and P value of hiatal hernia, erosive esophagitis, and Barrett’s epithelium according to different alcohol consumption levels. *logistic regression for analysis. **linear regression of logistic regression for analysis of dose-response trends.
